# Supplementary figures and images for: Mice lacking nucleotide sugar transporter SLC35A3 exhibit lethal chondrodysplasia with vertebral anomalies and impaired glycosaminoglycan biosynthesis
Source: PLoS One. 2023 Apr 13;18(4):e0284292. doi: 10.1371/journal.pone.0284292 (PMC10101523; doi:10.1371/journal.pone.0284292)

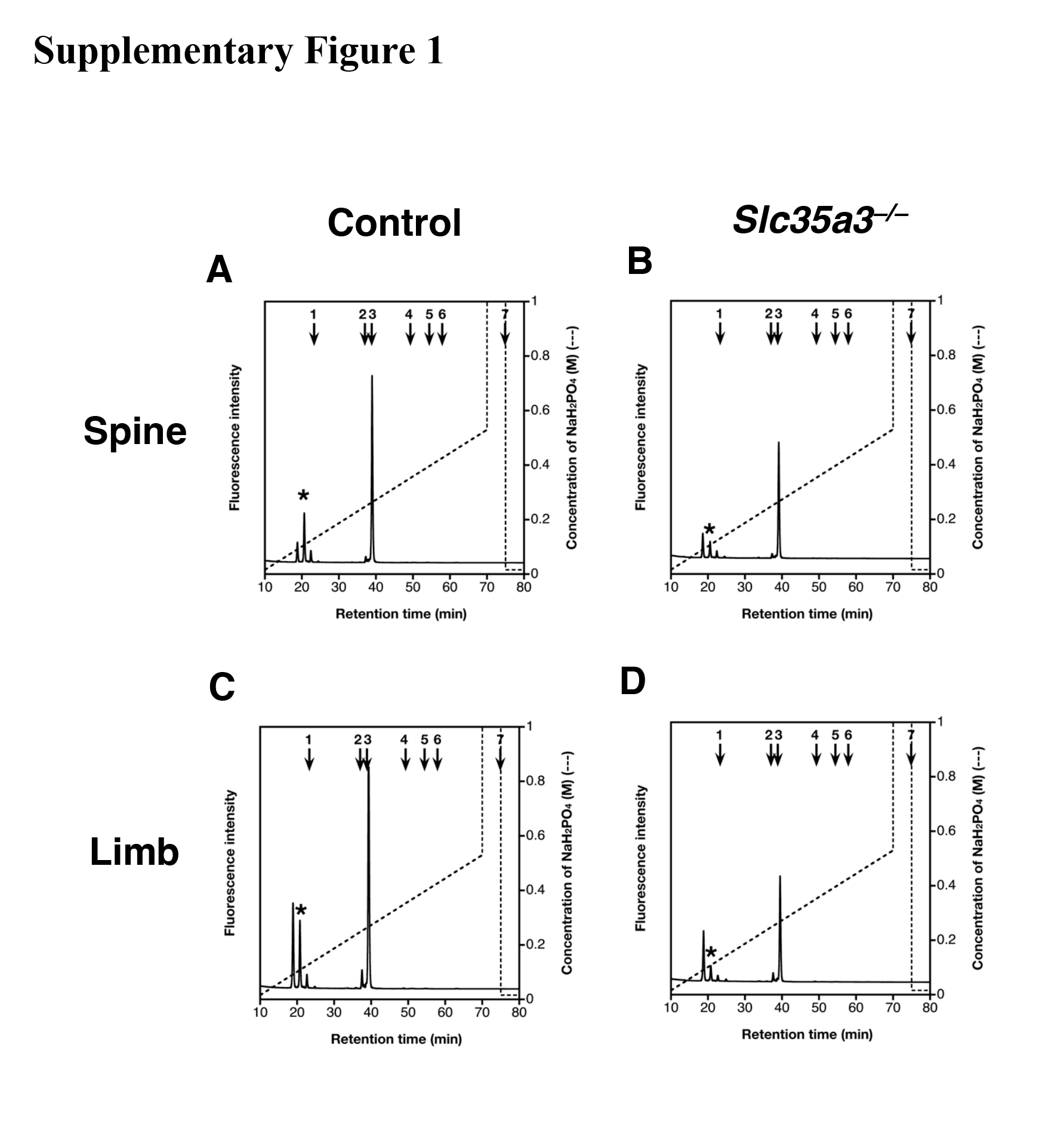

Supplement: S1 Fig — The 2-aminobenzamide (2AB)-derivatives of the yielded CS/DS and HA disaccharides after digestion with a mixture of chondroitinases ABC and AC-II, were separated using anion-exchange HPLC on an amine-bound silica PA-G column with a linear gradient of NaH2PO4 as indicated by the dashed line for the analysis of CS/DS and HA. HPLC profiles of the spine (A, B) and limb (C, D) samples of control (A, C) and Slc35a3−/− mice (B and D). The elution positions of 2AB-labeled CS/DS disaccharide standards are indicated by numbered arrows: 1, ΔHexUA-GalNAc; 2, ΔHexUA-GalNAc(6S); 3, ΔHexUA-GalNAc(4S); 4, ΔHexUA(2S)-GalNAc(6S); 5, ΔHexUA(2S)-GalNAc(4S); 6, ΔHexUA-GalNAc(4S,6S); 7, ΔHexUA(2S)-GalNAc(4S,6S). Asterisk indicates the ΔHexUA-GlcNAc derived from HA. Abbreviations: CS, chondroitin sulfate; DS, dermatan sulfate; HA, hyaluronan, ΔHexUA, 4,5-unsaturated hexuronic acid; GalNAc, N-acetyl-D-galactosamine; GlcNAc, N-acetyl-D-glucosamine; 2S, 2-O-sulfate; 4S, 4-O-sulfate; 6S, 6-O-sulfate. (TIF) [file pone.0284292.s001.tif]

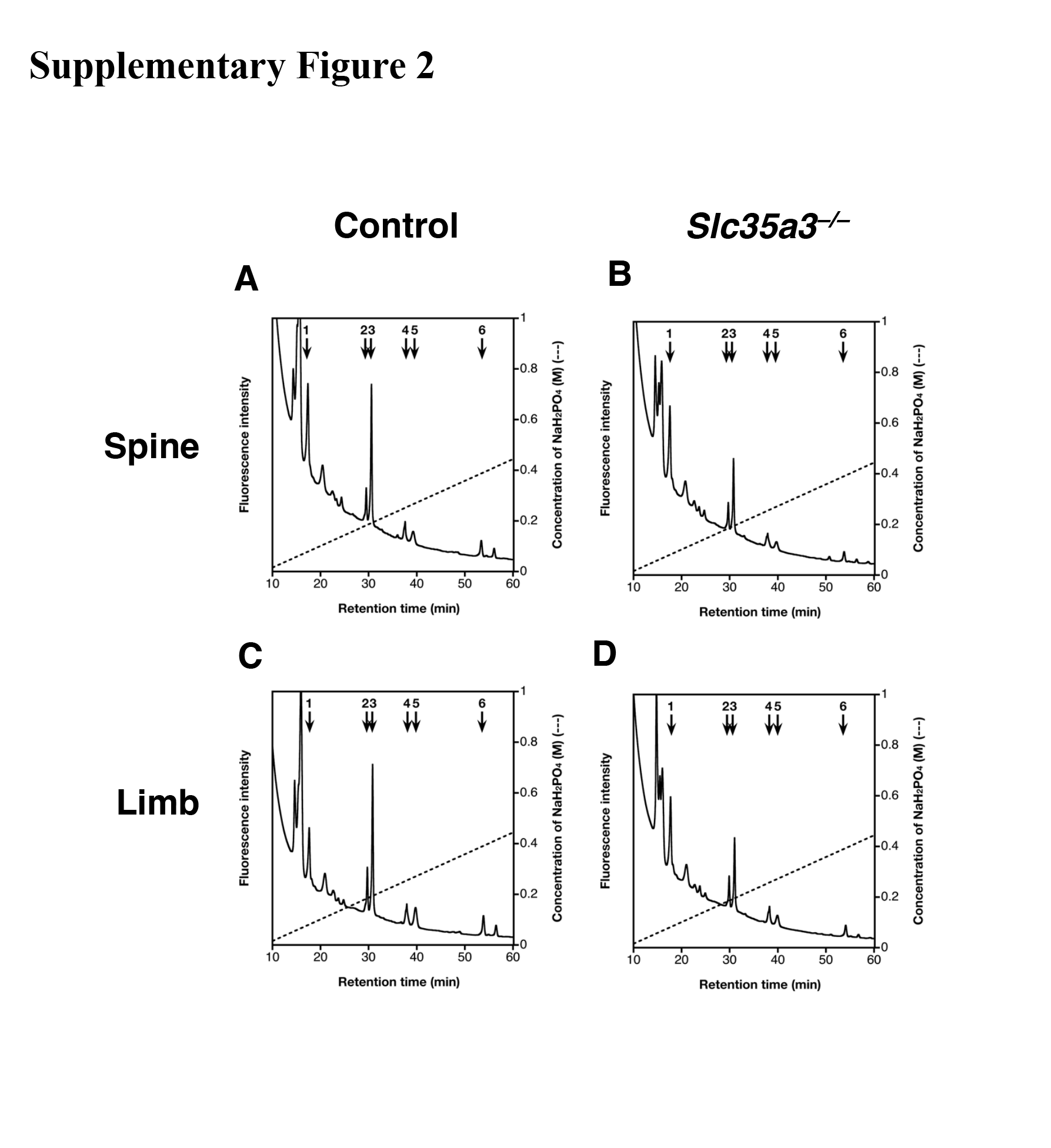

Supplement: S2 Fig — The 2-aminobenzamide (2AB)-derivatives of the yielded HS disaccharides after digestion with a mixture of heparinase-I, heparitinase-II, and heparinase-III, were separated using anion-exchange HPLC on an amine-bound silica PA-G column with a linear gradient of NaH2PO4 as indicated by the dashed line for the analysis of HS. HPLC profiles of the spine (A, B) and limb (C, D) samples of control (A, C) and Slc35a3−/− mice (B, D). The elution positions of 2AB-labeled HS disaccharide standards are indicated by numbered arrows: 1, ΔHexUA-GlcNAc; 2, ΔHexUA-GlcNAc(6S); 3, ΔHexUA-GlcN(NS); 4, ΔHexUA-GlcN(NS,6S); 5, ΔHexUA(2S)-GlcN(NS); 6, ΔHexUA(2S)-GlcN(NS,6S). Abbreviations: HS, heparan sulfate; ΔHexUA, 4,5-unsaturated hexuronic acid; GlcNAc, N-acetyl-D-glucosamine; GlcN, D-glucosamine; 2S, 2-O-sulfate; 4S, 4-O-sulfate; 6S, 6-O-sulfate; NS, 2-N-sulfate. (TIF) [file pone.0284292.s002.tif]

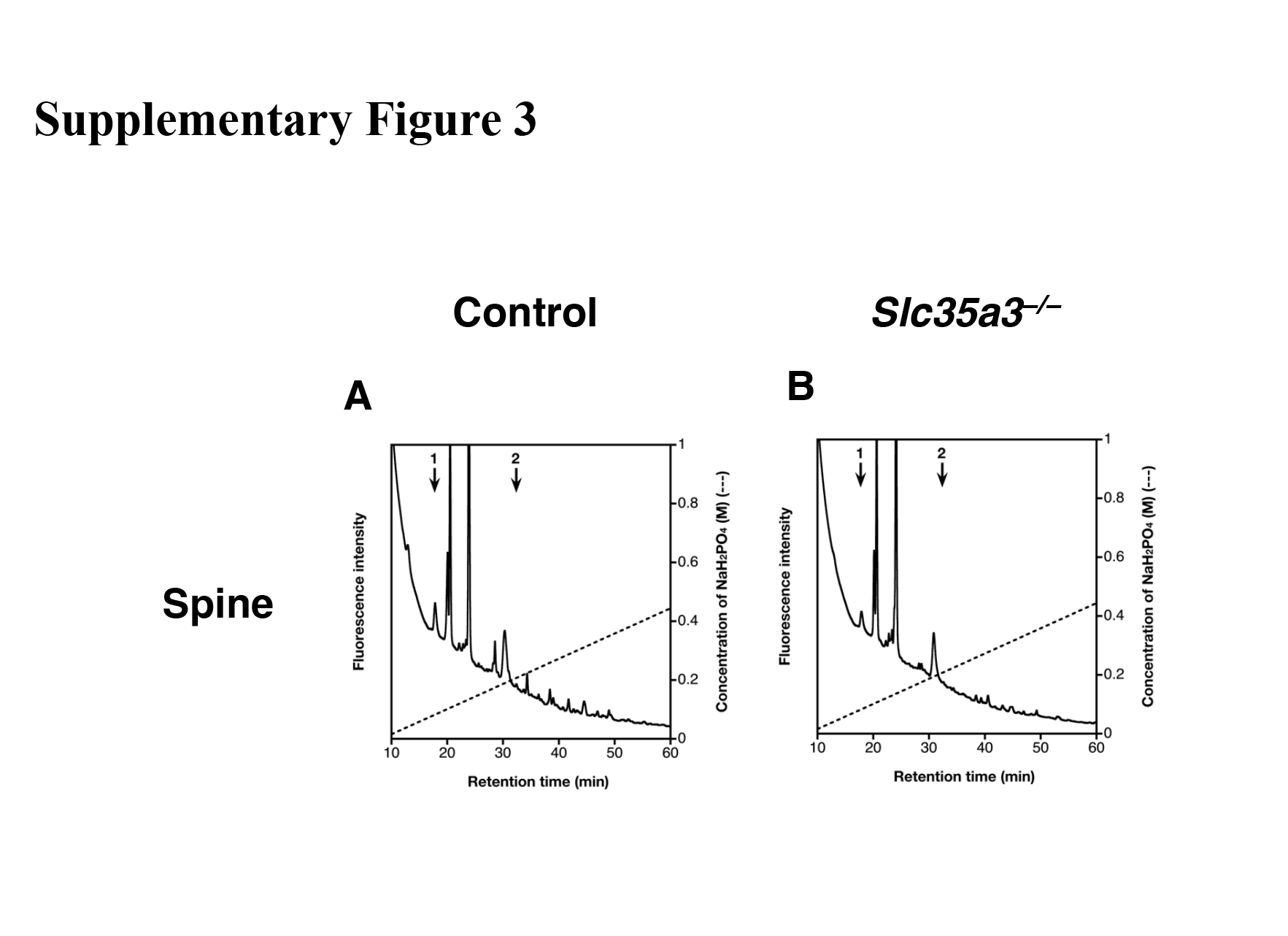

Supplement: S3 Fig — The 2-aminobenzamide (2AB)-derivatives of the yielded KS disaccharides after digestion with a keratanase-II were separated using anion-exchange HPLC on an amine-bound silica PA-G column with a linear gradient of NaH2PO4 as indicated by the dashed line for analysis of KS. HLPC profiles of the spine samples of control (A) and Slc35a3–/–mice (B). The elution positions of 2AB-labeled KS disaccharide standards are indicated by numbered arrows: 1, Gal-GlcNAc(6S); 2, Gal(6S)-GlcNAc(6S). Abbreviation: KS, keratan sulfate; Gal, D-galactose; GlcNAc, N-acetyl-D-glucosamine; 6S, 6-O-sulfate. (TIF) [file pone.0284292.s003.tif]

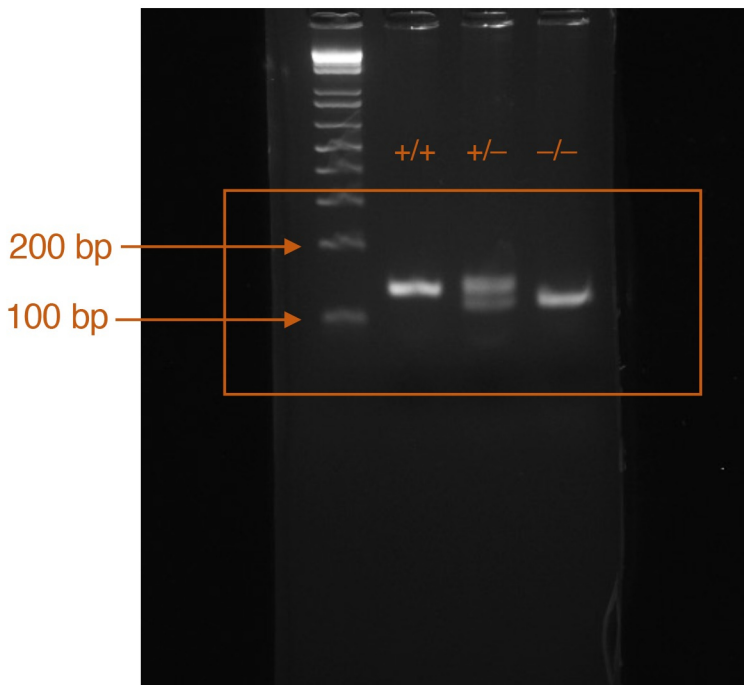

The raw image for Fig 1B

Supplement: S1 Raw images — (PDF) [file pone.0284292.s004.pdf]

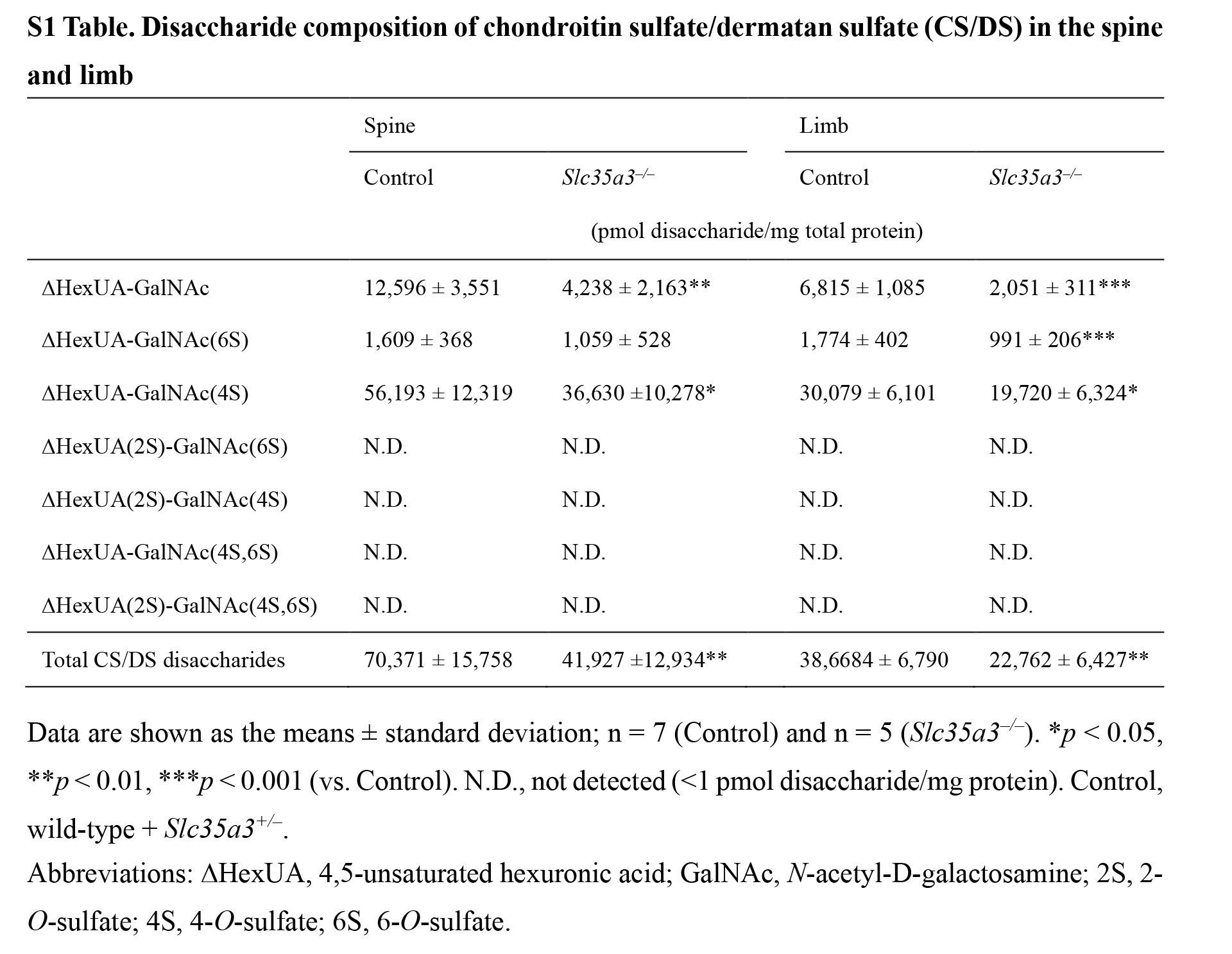

Supplement: S1 Table — (TIF) [file pone.0284292.s005.tif]

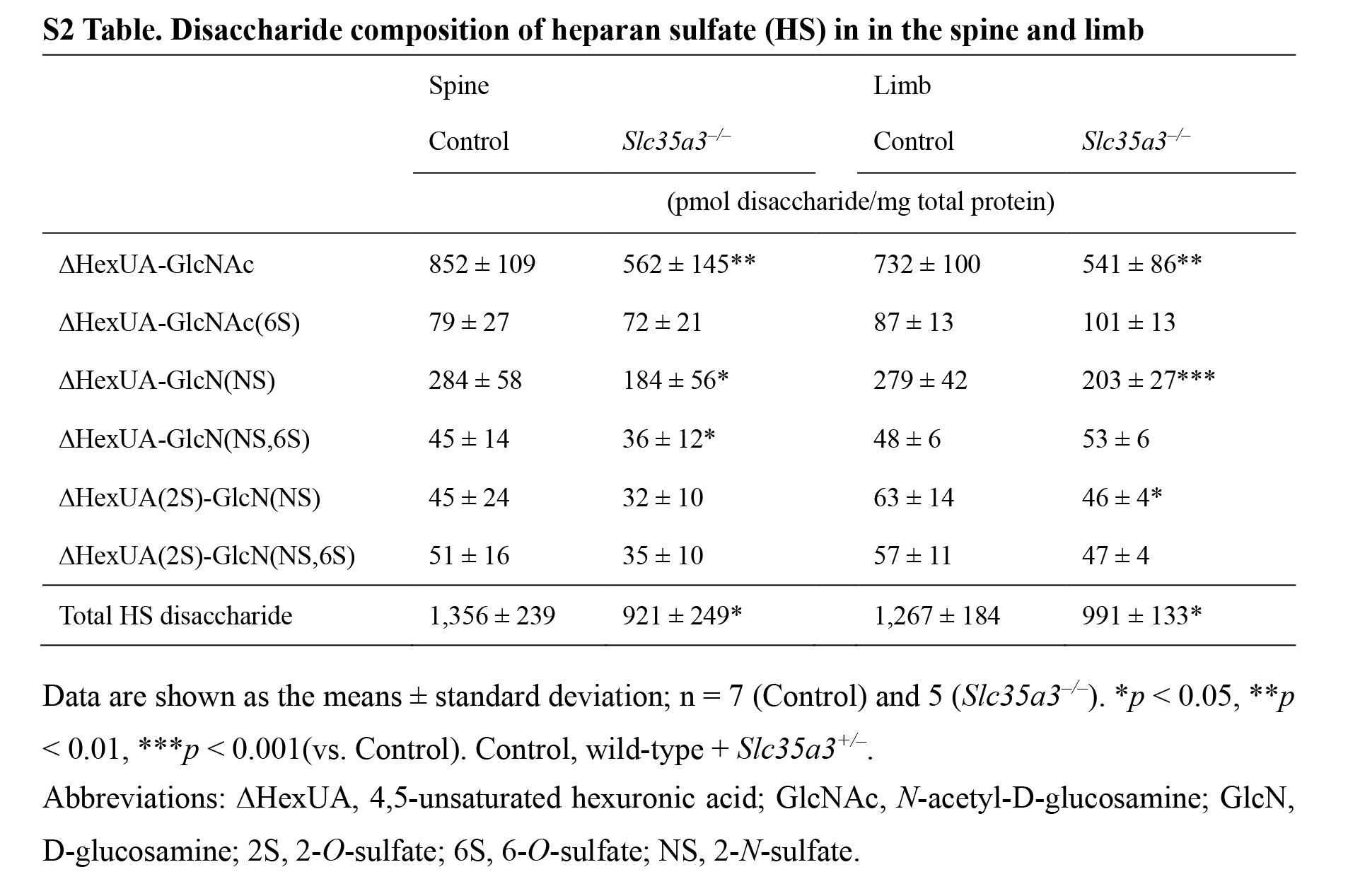

Supplement: S2 Table — (TIF) [file pone.0284292.s006.tif]

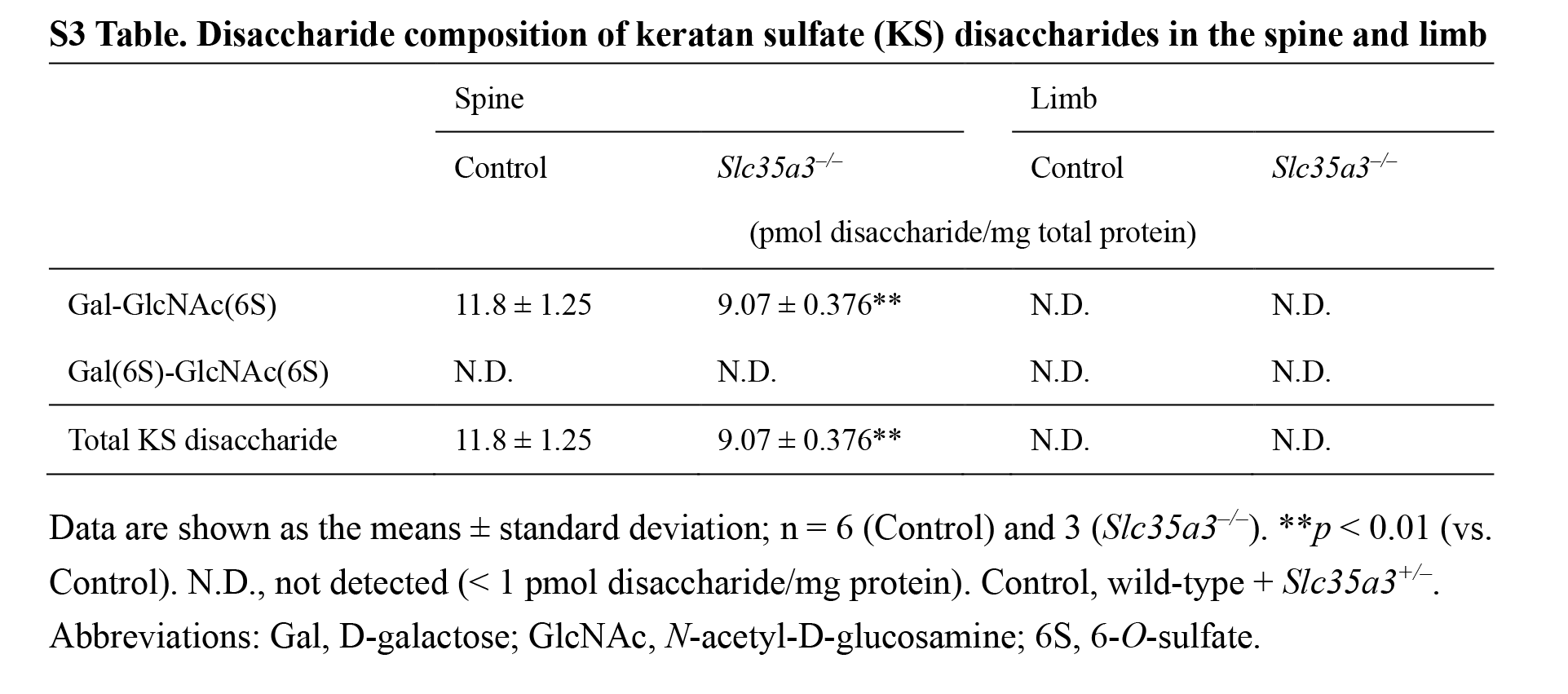

Supplement: S3 Table — (TIF) [file pone.0284292.s007.tif]

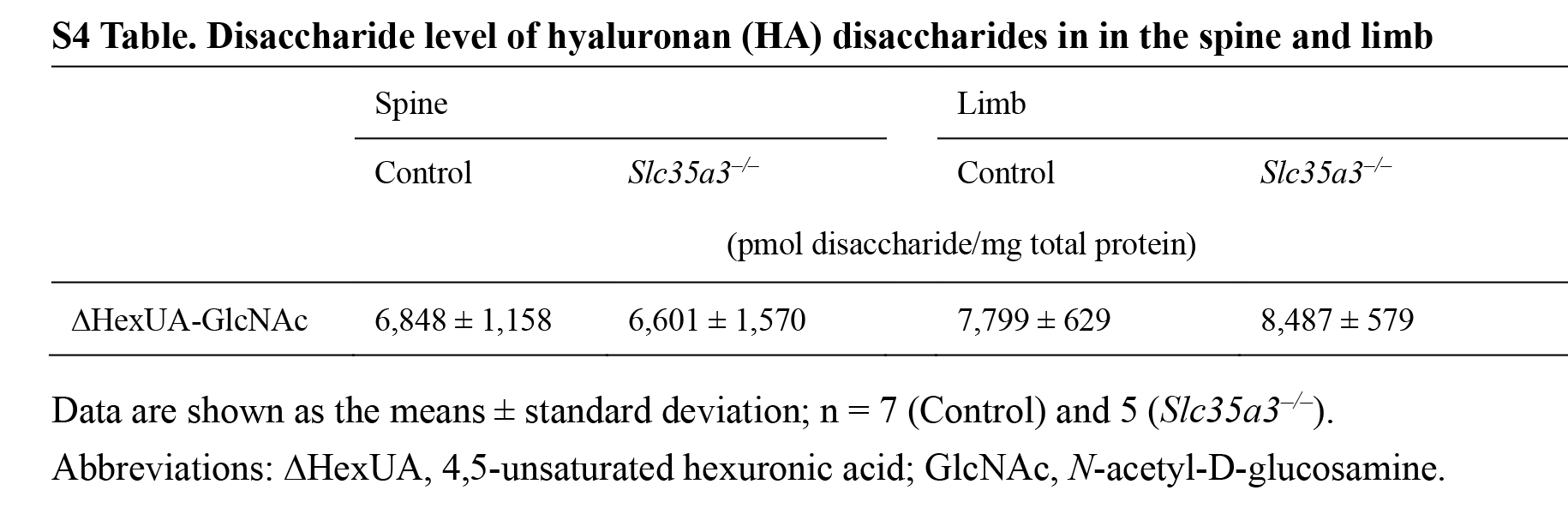

Supplement: S4 Table — (TIF) [file pone.0284292.s008.tif]
