# Supplementary material for: Mice lacking nucleotide sugar transporter SLC35A3 exhibit lethal chondrodysplasia with vertebral anomalies and impaired glycosaminoglycan biosynthesis
Source: PLoS One. 2023 Apr 13;18(4):e0284292. doi: 10.1371/journal.pone.0284292 (PMC10101523; doi:10.1371/journal.pone.0284292)
Supplement: Attachment — Submitted filename: Response to Reviewers.doc [file pone.0284292.s009.doc]

**<To Reviewer #1>**

**Comment:** In the results, GAG synthesis in cartilage from mutant and wt mice is measured by quantitation of disaccharides from different GAG species in nmol/mg total protein. However in the results and also in the Material and Methods session it is not specified at which step of the experiment total proteins have been measured and the assay which has been used.

**Response:** We added the following sentence in page 9: “Total amount of proteins in the sonicates was determined by micro BCA protein assay kit (Thermo Fisher Scientific)”.

**Comment:** I’d mention that the RC, FC and HC zones in Fig 4 and 5 point to resting, proliferative and hypertrophic zones of the growth plate.

**Response:** We described the RC, FC and HC zones in the Figure legends as following in page 14: High magnification images of the round chondrocyte (RC), flat chondrocyte (FC), and hypertrophic chondrocyte (HC) zones in the growth plates, RC (Fig 4 legend), round chondrocyte zone; FC, flat chondrocyte zone (Fig 5 legend).

**Comment:** There are some inaccuracies that should be checked:

Page 8. Line 4 from bottom “lumber vertebrae”

Page 12 line 10 from top “embryos et E18.5” and line 15 from top “lumber vertebrae”

Page 14 line 16 from top “Sic35a3-/-“

Page 15 lines 6 and 7 from top “lumber vertebrae”

**Response:** We corrected these errors in page 11, 12 and 14.

**Comment:** There are some inaccuracies that should be checked:

Page 18 line 11 from top “Slc35d-/-“

**Response:** The original is correct (page 18).

**<To Reviewer #2>**

**Comment:** Coming back to the GAGs analysis by HPLC after enzymatic digestions, how can the authors explain in their analysis (Supp figure 2) that a deficiency affects more the pick 3 corresponding to ∆HexUA-GlcN(NS) than the pick 2 corresponding to ∆HexUA- GlcNAc(6S)?? Is it a bias due to the specificities of the enzymes used to cleave the GAGs chains? This can be annoying.

**Response:** We thank reviewer#2 for the constructive comment. The greater reduction of ∆HexUA-GlcN(NS) than ∆HexUA-GlcNAc(6S) in *Slc35a3^–/–^* samples does not seem to be caused by the bias of HS-degrading enzymes, because the mixture of heparinases -I, -II, and -III can act equally on the HexUA-GlcN(NS) and HexUA-GlcNAc(6S)-containing sequences. The cause is unknown, but we would like to propose the following as one possibility. SLC35A3 is known to form huge complexes involved mannoside acetylglucosaminyltransferases (Mgats) and affect Mgat activities. Therefore, SLC35A3 may also form complexes with the HS-polymerase, a hetero-complex of EXT1 and EXT2, and affect their activities. The sugar backbone of HS, -GlcUA-GlcNAc-, is formed by EXT1/EXT2, and then *N*-deacetylase/*N*-sulfotransferase-1 (NDST1) catalyzes *N*-deacetylation and *N*-sulfation of GlcNAc residue in HS, resulting in the formation of HexUA-GlcN(NS)-containing sequence. EXT2 is reported to bind with NDST1 and regulate NDAT1 activity to form HexUA-GlcN(NS) (Presto, et al., Proc Natl Acad Sci USA 105: 4751-6, 2008). Therefore, SLC35A3 deficiency may cause not only depletion of UDP-GlcNAc but also the impairment of collaborative interaction of EXT1/EXT2 with NDST1 in the Golgi apparatus, which seems to lead to a decrease in conversion from HexUA-GlcNAc to HexUA-GlcN(NS). Consequently, the amount of HexUA-GlcN(NS) may be drastically decreased in *Slc35a3^–/–^* mice. In contrast, HS 6-*O*-sulfotransferase (HS6ST) catalyzes the transfer of sulfate to the C-6 position of GlcNAc residue in a non-cooperative way with EXT1/EXT2. Therefore, the conversion from HexUA-GlcNAc to HexUA-GlcNAc(6S) may not be severely affected in in *Slc35a3^–/–^* mice.

Currently, the investigation to identify multiprotein complexes involving SLC35A3 in cartilage is in progress, probably leading to the elucidation of this unclear mechanism.

**Comment:** I fully agree with a general GAGs defect from the experiments but the differential quantification defect on HS/ KS/ DS is much more difficult to assess from the experiments. This “strong” GalNAc defect is also interesting but stays descriptive. This is where O-GalNAc glycosylation analysis would be very interesting in confirming this defect.

**Response:** We are also interested in the *O*-linked fucose containing structures on Notch receptors in *Slc35a3^–/–^* mice. However, the endogenous *O*-linked fucose structures on Notch receptors could not be determined in mice because the *in vitro* glycosyltransferase assays using radiolabelling glycan technique and overexpression of the glycosyltransferases in CHO cell line that cannot synthesis complex or hybrid-type-*N*-glycans are essential to determine it (Moloney DJ et al, Nature 406: 369–, 2000). This paper focuses on analysis of the skeletal tissues (cartilage and spine) in *Slc35a3*^–/–^ mice. The amounts of *N-*glycan and *O*-glycan in skeletal tissues are very small; therefore, few papers reported the roles of *N*-glycans and *O*-glycans in skeletal tissues. SLC35A3 is reported to regulate the branching of *N*-glycans, and AMRS caused by *SLC35A3* mutations and congenital disorders of *N-*linked glycosylation are characterized by mental retardation and epilepsy. Therefore, we are interested in the interactions of SLC35A3 functions and *N*-glycan metabolisms in brain and have initiated the studies to examine them. It will take some time to obtain the results, and so we hope to report the results in our second paper. We have provided this information in page 19-20. We would like to take the interactions between SLC35A3 and *O*-glycan as the future consideration.

**Comment:** 2. The authors say that “the relationship between the function of SLC35A3 and GAG biosynthesis is largely unknown”. I do not agree with this sentence. The link has been evidenced in Haouari’s paper on serum bikunin isoforms in congenital disorders of glycosylation and linkeropathies. The clear defect observed on bikunin in SLC35A3 patient strongly demonstrates that GAGs are affected

**Response:** We accept this comment. " is largely unknown " was changed to " is not fully clarified" in page 5.

**<To Editor>**

**Comment:** To comply with PLOS ONE submissions requirements, in your Methods section, please provide additional information regarding the experiments involving animals and ensure you have included details on (1) methods of sacrifice for adult mice and mice embryos, (2) methods of anesthesia and/or analgesia, and (3) efforts to alleviate suffering.

**Response:** We added the following sentence in page 5: “Mice were sacrificed by an overdose of pentobarbital or cervical dislocation under isoflurane anesthesia. Mouse embryos were sacrificed by decapitation.”.

**Comment:** Please remove any funding-related text from the manuscript and let us know how you would like to update your Funding Statement. Currently, your Funding Statement reads as follows:

**Response:** We deleted the following in page 20:“This study was supported by Grant-in-Aid from the Ito Foundation (T.F.). This study is also supported in part by a Grant-in-Aid for Science Research (C) from the Japan Society for the Promotion of Science (JSPS) (21K0593219 to T.F.; 19K07054 to S.M.; 21K065520 to S.Y.), Grant-in Aid from the Research Center for Pathogenesis of Intractable Diseases from the Research Institute of Meijo University (S.M. and S.Y.), and Grants-in-Aid for Research from the National Center for Global Health and Medicine (20A1019 and 20A2007D to T.O.(Okamura); 21A1018 to K.N.).The funders had no role in study design, data collection and analysis, decision to publish, or preparation of the manuscript.”

**Comment:** In your cover letter, please note whether your blot/gel image data are in Supporting Information or posted at a public data repository, provide the repository URL if relevant, and provide specific details as to which raw blot/gel images,

**Response:** We provided the original image for Fig 1B in Supporting Information.
